# White Matter Hyperintensities among Older Adults Are Associated with Futile Increase in Frontal Activation and Functional Connectivity during Spatial Search

**DOI:** 10.1371/journal.pone.0122445

**Published:** 2015-03-20

**Authors:** Samuel N. Lockhart, Steven J. Luck, Joy Geng, Laurel Beckett, Elizabeth A. Disbrow, Owen Carmichael, Charles DeCarli

**Affiliations:** 1 Imaging of Dementia and Aging Lab, University of California Davis, Davis, CA, United States of America; 2 Department of Neurology, University of California Davis, Davis, CA, United States of America; 3 Neuroscience Graduate Group, University of California Davis, Davis, CA, United States of America; 4 Department of Psychology, University of California Davis, Davis, CA, United States of America; 5 Center for Mind and Brain, University of California Davis, Davis, CA, United States of America; 6 Division of Biostatistics, University of California Davis, Davis, CA, United States of America; 7 Department of Neurology, Louisiana State University Health Sciences Center—Shreveport, Shreveport, LA, United States of America; 8 Pennington Biomedical Research Center, Louisiana State University, Baton Rouge, LA, United States of America; Center for BrainHealth, University of Texas at Dallas, UNITED STATES

## Abstract

The mechanisms by which aging and other processes can affect the structure and function of brain networks are important to understanding normal age-related cognitive decline. Advancing age is known to be associated with various disease processes, including clinically asymptomatic vascular and inflammation processes that contribute to white matter structural alteration and potential injury. The effects of these processes on the function of distributed cognitive networks, however, are poorly understood. We hypothesized that the extent of magnetic resonance imaging white matter hyperintensities would be associated with visual attentional control in healthy aging, measured using a functional magnetic resonance imaging search task. We assessed cognitively healthy older adults with search tasks indexing processing speed and attentional control. Expanding upon previous research, older adults demonstrate activation across a frontal-parietal attentional control network. Further, greater white matter hyperintensity volume was associated with increased activation of a frontal network node independent of chronological age. Also consistent with previous research, greater white matter hyperintensity volume was associated with anatomically specific reductions in functional magnetic resonance imaging functional connectivity during search among attentional control regions. White matter hyperintensities may lead to subtle attentional network dysfunction, potentially through impaired frontal-parietal and frontal interhemispheric connectivity, suggesting that clinically silent white matter biomarkers of vascular and inflammatory injury can contribute to differences in search performance and brain function in aging, and likely contribute to advanced age-related impairments in cognitive control.

## Introduction

Older adults (OA) demonstrate interindividual differences in cognitive performance late in life even in the absence of clinical disease. This phenomenon of *cognitive aging* attributes individual cognitive differences, between otherwise “cognitively healthy” OA (i.e. free of clinical impairment), to differences in brain network architecture, particularly alterations in frontal cortical function, that are due to the aging process [1–6]. Additional research demonstrates that this cognitive and functional heterogeneity in older adults may be partly explained by individual differences between elders in the extent of white matter structural differences, differences that are themselves linked to clinically asymptomatic cerebrovascular disease (CVD) and inflammatory processes. Specifically, such white matter structural differences could impact the results of cognitive tasks and brain imaging methods among older adults [7–9], yet they are often not measured or controlled in such studies of healthy aging [8].

Structural white matter abnormalities called white matter hyperintensities (WMH) are known to increase with age, correlate negatively with deficits in processing speed, cognitive control, and visual search performance, and are associated with alterations (both increases and decreases) in brain functional activation and connectivity [8–15]. The underlying pathology of WMH is non-specific and includes demyelination, axonal atrophy, and gliosis [16], and WMH have been attributed to ischemic pathology and vascular processes [17] as well as to oxidative stress and inflammation [18]. Previous work suggests that WMH affect cognition through disruption of structural connectivity of distributed cortical networks necessary for specific functions, such as cognitive and attentional control [7,8], potentially independent of the effects of the aging process alone [9]. Among cognitively healthy elders, WMH exist throughout brain white matter (although there appears to be topographic specificity favoring periventricular regions [12]), and there is a significant relation between increased WMH volume, reduced frontal metabolism and impaired executive function [8,10,19]. A wealth of structural MRI and functional MRI (fMRI) literature has also shown that healthy older adults demonstrate reduced frontal white matter integrity, reduced anterior-posterior functional connectivity and white matter integrity, and greater bilateral recruitment of brain systems [20–23]. On the whole, these previous findings support a hypothesis whereby reduced frontal lobe white matter connections with network targets (potentially due to WMH) play a part in alterations in network functional activation and connectivity and cognitive performance commonly seen in healthy elders. Ignoring these factors, therefore, could risk attributing these WMH-related differences to the aging process alone.

For this study we sought to understand the importance of WMH volume to cognitive performance and brain function in healthy aging by examining how WMH are related to the function of a specific frontal-parietal cognitive network in healthy older adults, using a task-based functional activation and connectivity experiment. Specifically, we examined whether WMH are associated with blood oxygenation-level dependent (BOLD) fMRI activation differences between OA during performance of a cue-guided visual search task, a paradigm known to selectively engage frontal-parietal attentional control regions [24,25]. We additionally used a beta series correlation (BSC) approach [26] to explore associations of WMH volume with task-based attention network functional connectivity, to address whether WMH are associated with brain network communication and efficiency.

We specifically hypothesized that greater OA WMH volume (independent of chronological age) would be associated with reduced activation of attentional control network nodes. This would suggest that clinically asymptomatic white matter structural alterations are a significant contributor to brain network functional activation differences commonly ascribed to cognitive aging alone. We also examined, in selected activated attention network clusters, the WMH-related differences in the functional interdependence of regions during task performance using BSC, to address whether WMH are associated with brain network communication and efficiency. We predicted that, congruent with previous research, WMH would be associated with reduced functional connectivity [8]. We tested these hypotheses by having a sample of cognitively healthy elders (with a range of whole-brain WMH volumes) complete a visual search task during fMRI data collection, in order to examine how participant WMH volumes (independent of chronological age) were associated with task-based functional activation and connectivity.

## Materials and Methods

### Participants

Forty cognitively healthy OA participated (Table 1). OA aged 68–90 years in stable health were healthy controls from the UC Davis Alzheimer’s Disease Center Longitudinal Cohort, receiving detailed neuropsychological testing and clinical diagnosis of cognitively healthy or “normal” as described previously [27]. Participants performed standard neuropsychological tests to exclude participants with incident cognitive impairment; OA exhibited no clinically significant impairment. OA participants possessed whole-brain WMH volumes similar to the healthy aging population. All participants were right-handed, free of major illness, not taking medications thought to affect cognition, with normal or corrected visual acuity and color vision. None were excluded based on gender, race, or ethnicity. Procedures took ~3 hours with breaks as necessary. OA subjects who were non-compliant with task (*n* = 2), moved excessively (*n* = 2), or had a large subcortical stroke (*n* = 1) were excluded, yielding 35 OA for analyses (Table 1). Following region-of-interest (ROI) selection and functional connectivity data generation (below), one outlier OA with low connectivity values was removed, yielding 34 OA for functional connectivity analysis (results similar with outlier included). A power analysis was performed prior to the study to determine the extent to which our proposed sample size would be adequate to detect an effect of WMH volume on search task performance. It was concluded that 20 individuals would provide sufficient power (1-*β* > .80) for detecting effects of interest. We additionally recruited and tested 20 healthy young adults (YA) aged 20–30 from the University to aid in generation of region-of-interest masks for beta series correlation analyses (described below).

**Table 1:** Summary (Means and Standard Deviations or Frequencies) for Demographics, Neuropsychological Measures, and Brain Measures.

	OA (*n* = 35)
**Age** ^a^	78.1 (5.86)
**Years of education** ^a^	15.2 (3.29)
**Sex, F/M**	27/8
**% Minority** ^**b**^	57
**WMH**	-1.07 (1.06)
**MMSE score** ^a^	28.7 (1.26)
**Trails A time (s)** ^a^	35.2 (11.93)
**Trails B time (s)** ^a^	102.5 (45.99)

Note: WMH = White Matter Hyperintensities (corrected by total cranial volume, and log-normalized). MMSE = Mini-mental state examination.

^a^ Values displayed as *M* (*SD*) unless noted.

^b^ Minority defined as subject not identifying as White Non-Hispanic.

### Ethics Statement

The UC Davis Institutional Review Board and the UC Davis Alzheimer’s Disease Center executive committee approved the study. All participants were adults and provided written informed consent.

### Cognitive Control Task

**Fig 1:**
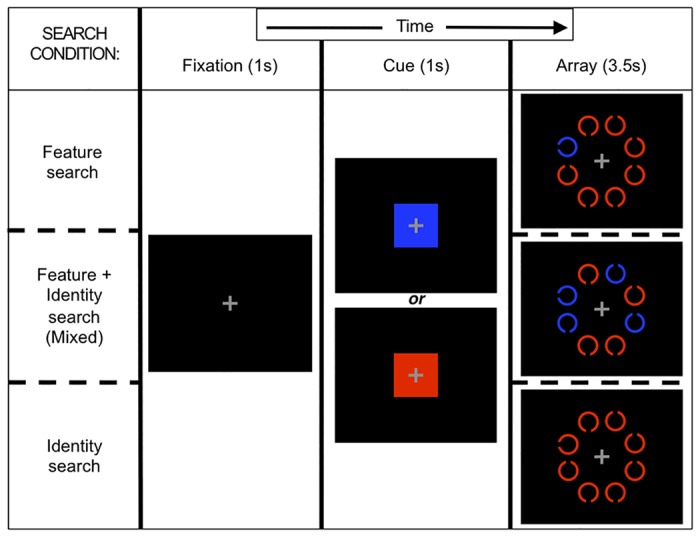
Cue-guided search task conditions. For each condition, an example 8-item array is presented with one possible target-distracter color combination.

Participants performed an in-scanner cue-guided search task (modified from [9]). In 3 search conditions (Feature, Mixed, Identity; see Fig. 1) different target/distracter contrast levels invoked varying levels of top-down and bottom-up attentional control across different overall processing demand levels [28,29]. Notable differences (following modification for scanner use) include a fixed but shorter duration array presentation, fewer colors, and fewer trials.

Stimulus presentation and response acquisition were controlled using Presentation (v14.9, neurobs.com) on Windows XP. Participants, using left or right index finger button presses on a single response box, were to quickly and accurately report which of two targets (left- or right-oriented C, each 50% of trials) was present in a set of distracters (up- and down-oriented C’s) while reaction time (RT) was measured. A central fixation cross began each trial, with variable interstimulus interval durations averaging 4000 ms (>2000 ms) calculated using OptSeq (surfer.nmr.mgh.harvard.edu/optseq), followed by a color cue indicating target color (1000 ms), then the search array (3500 ms). Using a mirror, stimuli were viewed on a black background, projected on a screen (2” diameter per C) at the participant’s feet. Red and blue cue/stimulus pairs were each presented randomly on 1/2 of trials.

In Feature search, the target appeared in the cued color and any present distracters were in the remaining color; this condition elicited minimal top-down control as the target attracted attention automatically, and bottom-up signaling primarily drove search [28]. In Mixed search, half the items were in the cued color (always including target) with the other half (distracters) in the remaining color; a working memory representation could be used to eliminate items, but individuals with poor top-down control will search uncued items, leading to slowed RTs [29]. In Identity search, all items were in the cued color, and target detection relied upon top-down-controlled search processes, as no salient features distinguished the target.

We varied array size (1, 4, 8 items) to measure fMRI activation by set size. Task presentation was blocked by condition, with size randomly determined per trial; each block was divided into 2 counterbalanced mini-blocks to provide rest and limit length. There were 32 trials, for 9 combinations of set size and search condition (hereafter, *cells*), for 288 trials per participant. Participants practiced the task prior to the experiment; none complained of difficulty.

For analyses, we removed incorrect response trials, excluded trials with RTs <200 ms as anticipation responses, and excluded as outliers trials with RT >3 SD from each subject’s cell mean, yielding *trimmed correct trials* [9].

### MRI Acquisition and Processing

#### Brain MR sequences

Brain MRI data were obtained on a 3T Siemens TIM Trio scanner with an In-vivo Inc. 8-channel head coil. High-resolution T1-weighted ([repetition time] TR/ [echo time] TE: 2000/2.91 ms, slice thickness: 1.0 mm, 208 slices, field of view [FOV]: 256 X 256 mm, matrix: 524 X 524) and FLAIR (TR/TE/[inversion time] TI = 9000/91/2250 ms, slice thickness: 2.0 mm, 70 slices, FOV: 204 X 256 mm, matrix: 524 X 524) sequences were acquired. We acquired 2 gradient-recalled echo (GRE) field map images (1 with head coil [GRE-HC], 1 with body coil [GRE-BC], with identical parameters: TR/TE1/TE2: 415/2.46/4.92 ms, 64 slices, slice thickness 4.0 mm, FOV 256 X 256, matrix: 64 X 64) to correct for B0 and B1 effects. We acquired 6 (7:16 duration) sessions of T2-weighted echo planar BOLD functional images during task performance (TR/TE: 2000/25 ms, 34 slices, 3.4 mm slice thickness, FOV: 220 X 220 mm, matrix: 128 X 128, flip angle: 90°).

#### Intensity corrections

We used GRE TE1 magnitude images for correction of spatial variability in head coil sensitivity, which produces tissue intensity variability in participant T1 and FLAIR images. In separate pipelines for T1 and FLAIR, we aligned and resampled GRE images to the anatomical image, divided the GRE-HC by the GRE-BC image to generate a head coil sensitivity map, then divided the anatomical image by the sensitivity map yielding an intensity-corrected anatomical image.

#### WMH segmentation

Total intracranial (TCV) and WMH volume were measured from intensity-corrected FLAIR images, by operators blind to participant age and gender, using the in-house semi-automated Quanta software package and analysis protocol that have been previously described [9,12,30,31,32]. Non-brain elements were first manually removed from images by operator-guided tracing of dura mater within the cranial vault—including middle cranial fossa, but excluding posterior fossa and cerebellum. The volume of this traced region was defined as TCV, and tissues outside the TCV were removed from the image. Image intensity nonuniformities were then removed from the image, and the corrected image was modeled as a mixture of Gaussian probability functions corresponding to brain and to non-brain tissue, with the segmentation threshold between brain and non-brain intensities located at the minimum probability between these two distributions. The volume of voxels on the brain side of this threshold was then classified as brain volume. We then applied morphometric erosion of two exterior pixels to brain volume voxels, in order to remove the effects of partially volumed CSF voxels during WMH detection. We then fit a single Gaussian distribution to the intensity distribution of remaining brain volume voxels, and all voxels with intensity >3.5 SD above the mean brain volume intensity were defined as WMH.

A rigorous protocol ensured cross-analyst validity [9]. We corrected for head size by expressing WMH volume as percentage of total intracranial volume, and log-transformed for normality, yielding log-normalized WMH volumes (lnWMH) values, which were z-transformed for statistical modeling.

#### FMRI processing

We discarded the 1st 4 functional image TRs of each session to let signal reach steady-state, concatenated across time within subject, then performed canonical fMRI preprocessing using SPM8 (fil.ion.ucl.ac.uk/spm), following closely as possible previous publications from our lab (e.g., [8]). We conducted slice-timing correction (sinc interpolation, ascending-interleaved even-first slices, middle time-point reference). Using gradient-echo field map images we generated voxel displacement maps (SPM8 FieldMap toolbox) to enable within-subject realignment and head motion correction, motion parameter generation, and unwarping. We normalized each session’s mean image to the Montreal Neurological Institute (MNI) EPI.nii template image, (MNI152 space), and applied these transformations to all images for each session, using parameters specified in the SPM manual (affine regularization: ICBM/MNI template, nonlinear frequency cutoff: 25; nonlinear iterations: 16; nonlinear regularization: 1; preserve concentrations, trilinear interpolation, no wrapping). Following normalization we spatially smoothed using a Gaussian 8mm FWHM kernel before entering images into analyses.

There exists debate regarding appropriate use of standard (i.e., those recommended in SPM’s manual) or non-standard fMRI preprocessing pipelines in the cognitive neuroscience of aging. For example, while using standard preprocessing and analysis methods, including normalization of older adult brains to canonical templates defined using young brains, has been criticized [33,34], work using more traditional approaches allows greater comparability to past results within [8] and across [35] labs. One study [35] using SPM8 normalized their subjects (which included older adults of interest and younger adults) to the standard MNI152 EPI template; this was highly similar to the current study regarding functional image preprocessing methods. There are additional examples of older adult functional imaging studies using normalization to the standard MNI EPI template [36,37]. As coordinate locations of results in MNI space are well understood from one lab to another, we present our imaging results in MNI space in this manuscript.

With such debate, however, a combination of approaches to validate functional imaging results is warranted. Here, in addition to conducting a more traditional SPM analysis (using the canonical EPI template), we also performed functional image preprocessing for our older adult functional activation study using a study-specific EPI template generated in SPM8, following guidelines of [38], to confirm that our results were not an artifact of MNI152 template space. The goal of this approach was to create a study-specific and age-appropriate template based on the mean of individually normalized EPI images for use in group data analyses. The first EPI volume for each older adult subject was spatially normalized to the MNI EPI template (using the parameters described above), smoothed with 8mm FWHM Gaussian kernel, and then resulting EPI volumes were averaged over the subjects to form the study-specific template of this experiment, which was used for normalization and subsequent processing of functional images as described below for the traditional MNI EPI template (study-specific template results, are presented in S1 Fig.).

### Behavioral Study Statistical Modeling

We recapitulated behavioral analyses from an earlier report using the same task [9] although certain parameters (and inclusion in models) were modified for scanner use. We log-transformed RTs of *trimmed correct trials*, yielding *trimmed correct lnRTs*; slower lnRT indexed impaired search performance. As logarithmic transformations convert multiplicative into additive factors [39–41], we could examine the impact of independent variable interactions, which when observed using lnRT as a dependent variable, cannot be explained by generalized slowing.

We previously developed search contrasts representing hypothesized tests for trial lnRT differences (described below relative to fMRI hypotheses: *set level*, *set nonlinearity*, *condition difficulty*, and *selective vs*. *nonselective*). Contrasts treated Feature search and set size 1 as reference levels, controlling for subjects’ baseline perceptual processing speed and allowing comparison of different predictors’ effects on basic processing speed and more complex cognitive control. Fitting multivariate linear mixed-effects regression models [42] using *nlme* routines in R 3.1 [43] we modeled single-trial trimmed correct lnRTs among OA and associations with predictors including age and WMH volume. We centered OA education at 12 years and age at 65 years. Nuisance covariates were added stepwise and retained if *p* <. 1. To estimate OA age-performance relations we modeled OA single-trial trimmed correct lnRTs, with random effects of participant, and fixed effects of chronological age, search task contrasts, and all 2-way age*contrast interactions. To test whether WMH or other variables predicted OA performance we modeled single-trial trimmed correct lnRTs, with random effects of participant and fixed effects of the search task contrasts, WMH volume, and set level*WMH, condition difficulty*WMH, and set level*condition difficulty interactions. We specifically tested effects of WMH on modifying performance in difficult task scenarios with high cognitive control demand (larger set sizes; Mixed and Identity) relative to easier references (set size 1; Feature search). To assess the independent age and WMH contributions to the WMH-search relation, we constructed an identical model, adding age.

### FMRI Univariate Activation Study Statistical Modeling

For these analyses we restricted data to only *correct trials* (mean accuracy 96% for OA). Using motion vectors from within-subject registration, we identified all TRs where significant motion events occurred (>2° rotation or 2mm translation, any direction). We calculated which trials high-motion TRs contributed substantially to estimated betas (>1% total area under curve using SPM8’s canonical hemodynamic response function; TR +/- 11 TRs, up to 23 TRs per artifact), removing these trials from subsequent analysis (1.03% of OA trials).

For remaining (trimmed, correct, motion-scrubbed) trials we modeled trial RT duration convolved with SPM8's canonical hemodynamic response function. Relevant to fMRI analyses were search contrasts studied in behavioral analyses [9]. *Set level* tested for linear trends in activation with increasing set size, reflecting recruitment of additional top-down attentional control with more distracters. *Set nonlinearity* assessed nonlinear activation differences with increasing set size. *Condition difficulty* assessed whether activation was different in difficult conditions (Mixed and Identity) relative to the Feature condition (which was more reliant on bottom-up signaling). *Selective vs*. *nonselective* compared activation on Mixed and Identity conditions.

Voxel-level analyses compared activation differences by search contrast. Clusters were labeled as significant if passing a *p* <. 001 uncorrected voxel height threshold with k = 10 voxel cluster extent, a level selected based on analyses and reports on simulations balancing type I and II errors using combined voxel- and cluster-level thresholds, as neuroimaging research is charged with having high type II error rates ([44], see also [45–48]). We identified the profiles of significant search contrast-related activation differences for OA.

We further investigated, for each contrast separately, whether OA WMH volume was associated with search contrast activation profile differences. In this WMH-activation analysis, for each contrast we created separate masks of brain voxels representing significant OA group activation (*p* <. 001 uncorrected height threshold, k = 10 extent threshold) to examine differences in activation within specific cognitive networks associated with WMH. At each voxel within contrast-specific masks, we tested whether each OA subject's search contrast parameter value was significantly associated with WMH volume (independent of age, sex, and education), using a threshold suitable for smaller test regions (*p* <. 005 uncorrected height threshold, k = 10 extent threshold [44]). For example, for *set level*, this analysis demonstrates the location of subject-specific WMH-associated differences in activation increases with set size.

### FMRI Functional Connectivity Study Statistical Modeling

#### Univariate activation analysis and ROI identification

Functional connectivity analysis involved two stages, first identifying activation ROIs from an all-subjects univariate activation analysis, then correlation analysis of trial-specific beta values pairwise across ROIs (BSC analysis). We included a sample of 20 healthy young adults (processed exactly as the OA were processed) in this ROI definition step in order to minimize the chance that our ROI generation would be limited or biased by the age of the OA sample. We used every trimmed, correct, motion-scrubbed trial for every cell (up to 92 trials per set size and 288 trials per participant), for all YA and OA subjects, both to define ROIs and to test functional connectivity. We employed *set level*—which tested for linear activation trends with increasing trial set size (collapsed across condition), and which generated significant results for behavioral [9] and functional analyses—to define ROIs. In parallel with univariate analyses described above, in voxel-wise group-level analyses we identified regions showing significant activation increases with increasing set size (*set level*) for all subjects (YA and OA; *p* <. 001 uncorrected voxel height threshold, k = 10 extent threshold). The initial all-subjects univariate results (see “Univariate activation analysis and ROI identification,” below) confirm an expected activation profile (frontal, parietal, visual and thalamic clusters; e.g. [49,50]), supporting our hypothesis that subjects are engaging attentional control and strongly activating the frontal-parietal top-down attentional control network. We then focused functional connectivity analysis to activation ROIs within this attention network specific to top-down attentional control in aging [51,52] by masking by MarsBar v0.42 AAL atlas-derived regional masks for left and right middle frontal gyrus (LMFG, RMFG) and superior parietal gyrus (LSPG, RSPG), to generate 4 clusters for ROI-based functional connectivity analysis across all subjects.

#### Functional connectivity analysis

For BSC analyses [26], for OA subjects we first modeled each trimmed, correct, motion-scrubbed trial, yielding up to 288 whole-brain trial-specific beta estimate maps per subject, one per included trial (nuisance covariates included motion and block). Each beta map had an associated condition, set size, and order. Resulting trial beta maps were concatenated into a 4-D image (a *beta series*), and for each trial, betas within clusters (LMFG, RMFG, LSPG, RSPG) were averaged. Quality control analyses confirmed that beta distributions were normal. We calculated pairwise correlations between all cluster pairs to generate 6 BSC values (LMFG-RMFG, LMFG-LSPG, LMFG-RSPG, RMFG-LSPG, RMFG-RSPG, LSPG-RSPG). Pairwise BSCs were analyzed to identify differences in pairwise attention network functional connectivity by OA WMH volume. In group-level analyses, we used ANOVA to examine relations of independent variables, either age group or OA WMH volume, with dependent variables (BSCs of cluster pairs). Finally, to examine the role of functional connectivity in cognition in OA, we assessed relations between WMH, functional activation and connectivity, and performance.

## Results

### Participant Characteristics

OA demographics and neuropsychological scores are presented in Table 1. Whole-brain WMH volumes among OA were similar to the larger cognitively healthy aging population indicating good representation [53]. Amongst OA, there was no significant age-WMH association (*R* ^*2*^ = .08, *p* = .09).

### Visual Search Behavioral Study Results

**Fig 2:**
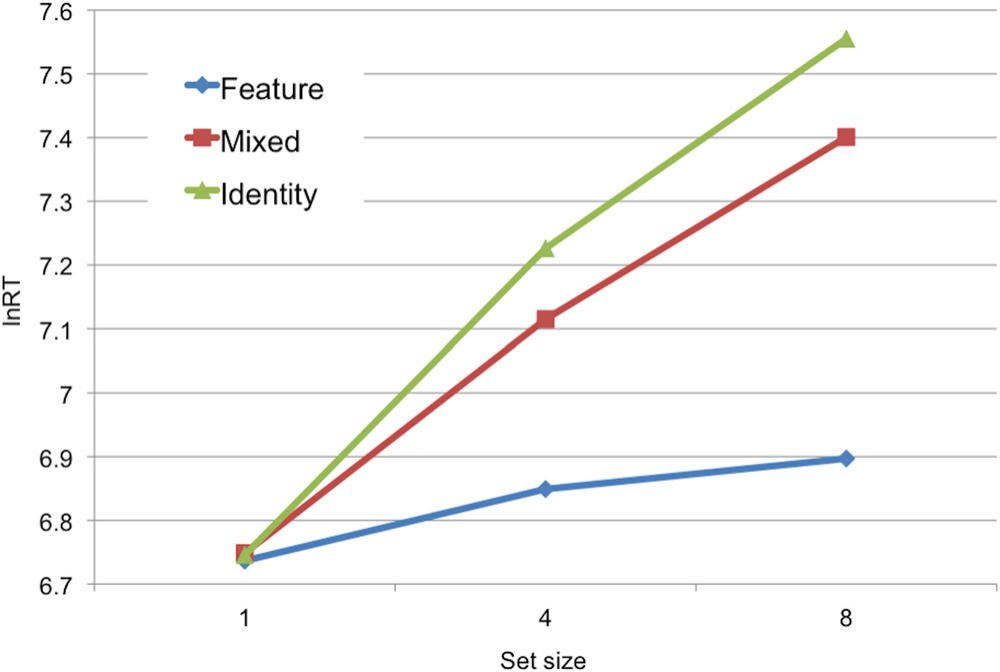
Mean trimmed correct log-normalized reaction time values. Values are plotted for each search condition and set size, for older adults.

When examining behavioral data as in our previous work (where performance was outside an MRI), results were largely concordant [9]. Overall accuracy was high (96% for OA), with longer lnRT was associated with more errors in OA (*R* ^*2*^ = .25; *p* <. 001), confirming our results were not confounded by speed-accuracy trade-off. Comparable to previous results [9,51,54], increasing task difficulty (set size or search condition) was associated with increasing lnRT (condition, set size, and condition*set size *p*'s <. 001), shown in Fig. 2.

OA age positively correlated with education (*R* ^*2*^ = .16, *p* = .018), contrasting with our previous study [9]. OA age was not associated with other measures that may influence performance (e.g., sex: *p* = .16), or with nuisance variables (e.g., target color: *p* = .86). OA chronological age was associated with search performance (slower lnRT, *p* = .0089). In contrast with our previous finding that age was not significantly associated with lnRT, age among OA plays a larger role here; therefore we carefully examined additional influences of chronological age and education in models below.

Increased OA WMH volume was associated with longer lnRT (*p* = .0237), again demonstrating that WMH are associated with impaired performance. There were significant search contrast main effects (all *p* <. 05) and a WMH*condition difficulty interaction (*p* = .0175). WMH remained significant (*p* = .048) after covarying for chronological age (age not significant, *p* = .40), suggesting that subject age—while present as a predictor itself—does not strongly modify the WMH effect on OA performance, as found previously, and suggesting that WMH impair cognitive control and performance independent of decreasing perceptual processing speed. To further scrutinize these results, we examined search contrasts associations with brain activation, and how activation differed by WMH.

### FMRI Activation Study Results

#### OA attention network activation

When examining voxel-level effects of search contrasts on activation, we found OA attention network activation for all contrasts; however, as our univariate activation and functional connectivity analyses were focused on *set level*, we will focus on these results.

**Fig 3:**
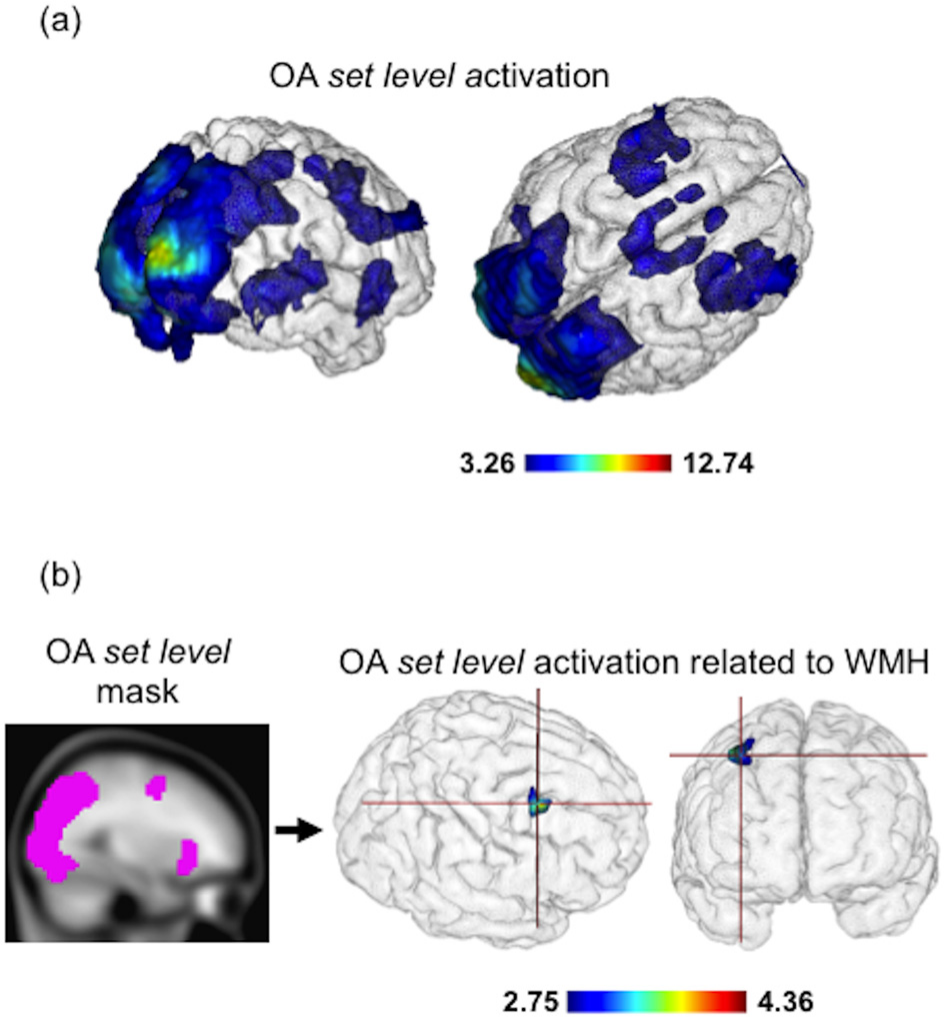
FMRI Activation results by age group and WMH volume. (a) Activation for older adults for *set level* search contrast; clusters labeled as significant if passing a *p* <. 001 uncorrected voxel height threshold with 10 voxel cluster extent. (b) *Set level* activation significantly associated with white matter hyperintensity volume among OA; clusters labeled as significant if passing a *p* <. 005 uncorrected voxel height threshold with 10 voxel cluster extent. The significant cluster (28 voxel extent, peak voxel Montreal Neurological Institute coordinates = 36, 5, 52) is located in right middle frontal gyrus (Brodmann area 6).

Examining *set level* (Fig. 3a; S1 Table), OA showed activation increases with linear set size increases over large spatial regions, particularly in frontal-parietal regions. OA showed activation increases across a network of visual, frontal, parietal, and thalamic regions; this *set level* network includes components of the dorsal (superior posterior parietal and frontal cortex) and ventral (inferior frontal nodes) attention systems. Significant clusters are also found in OA in visual regions in occipital, parietal and temporal cortex, extending toward clusters in the superior parietal lobule at or above the intraparietal sulcus. Clusters exist at the intersection of the precentral and middle frontal gyri, representing dorsal attention network nodes such as the frontal eye fields. These *set level* results were particularly strong, additionally passing a false discovery rate-corrected *p* <. 05 (k = 10 cluster extent) threshold.

Overall activation for the remainder of contrasts was generally limited. For *set nonlinearity*, OA showed activation increases with nonlinear set size increases in temporoparietal junction regions. For *condition difficulty*, clusters in ventral frontal-parietal attention regions (i.e. ventral frontal and temporoparietal cortex) were active for OA. For *selective vs*. *nonselective*, anterior frontal cortex clusters showed activation for OA for Identity relative to Mixed search.

#### WMH-related differences in attention network activation by contrast

Next, we investigated for each contrast whether OA WMH volume was associated with activation differences. Only *set level* yielded a significant result; in this contrast, there was a significant cluster of voxels in right middle frontal gyrus (RMFG) for which increased activation with increasing set size was significantly associated with brain WMH volume extent (i.e. *set level*-related over-activation; Fig. 3b). In the peak of this WMH-RMFG cluster, a 1 SD increase in log-normalized WMH volume (0.643% WMH volume increase relative to head size on average) was associated with a 15.1% increase in activation.

#### BSC Results

**Fig 4:**
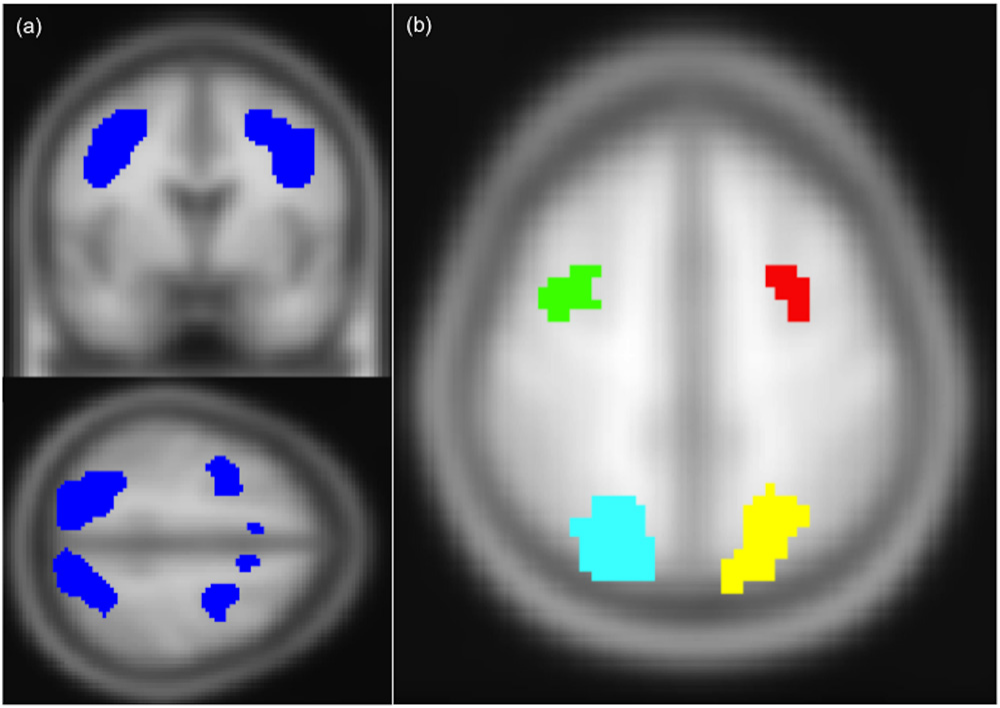
Statistical mask and regions-of-interest used as clusters in correlation analyses. (a) Mask generated from statistical analysis of *set level* contrast across young and older adult subjects. (b) Cluster regions-of-interest used in correlation analysis: right middle frontal gyrus (MFG; green, 1.674cc), left MFG (red; 1.215cc), right SPG (cyan, 5.292cc), left superior parietal gyrus (SPG; yellow, 6.912cc).

As discussed in the statistical modeling section, we developed our BSC ROIs using activation patterns from the *set level* contrast for old and young subjects combined (graphically displayed in Fig. 4a). From this ROI, we extracted 4 functionally independent regions known to be involved with spatial search based on previous results (Fig. 4b; see [51,52]). OA had positive correlations between all regions (all average *r*’s >. 55, mean *r* = .6696). OA specifically showed high (>. 7) interhemispheric, homologous correlations (i.e., left-right frontal and parietal correlations are high), with lower frontal-parietal correlations. BSC did not vary significantly by condition or set size.

#### WMH-related differences in network connectivity

We next explored whether OA WMH volume contributed to attention network functional connectivity. Only frontal interhemispheric BSCs were associated with WMH volume (Table 2), with greater OA WMH volume associated with higher LMFG-RMFG correlations (*p* = .0115), although it should be noted that this relation is above a multiple comparisons-corrected threshold of *p* < 0.0083. This relation also remained similar with age and education included in models.

**Table 2:** OA WMH Volume Differences in Frontal Interhemispheric BSC.

Predictor	*F*	*p*
**Set size**	0.6218	.5377
**Condition**	0.5503	.5774
WMH volume*	6.4655*	.0115*
Set size * Condition	1.0994	.3571
Set size * WMH volume	0.0062	.9938
Condition * WMH volume	2.7658	.0646
Set size * Condition * WMH volume	0.2321	.9202

Note: BSC = beta series correlation.

* Denotes significant at *p* <. 05.

### Relations between WMH, Search Performance, and Function

Given our finding that, among OA, increased WMH volume predicted poorer search performance (longer lnRT), increased RMFG cluster activation (WMH-RMFG cluster), and a trend toward increased task-based frontal interhemispheric BSC (“LMFG” and “RMFG” clusters), we hypothesized that increased activation might be associated with poorer performance. Given the finding of increased correlations between frontal regions in OA, we additionally hypothesized a relation between increasing extent of frontal BSC and poorer performance. To test these hypotheses, we examined associations between OA cluster activation, BSC, and performance. Using models similar to the WMH-performance model above, both greater WMH-RMFG activation (*p* = .046) and greater frontal interhemispheric functional connectivity (*p* = .031) were separately associated with slower search performance (lnRT).

We then sought to better understand the relation between WMH, search performance and frontal interhemispheric BSCs, by analyzing the relation between WMH-RMFG cluster activation and frontal interhemispheric functional connectivity. There was a strong positive association (*p* = .001) for WMH-RMFG independent of task difficulty (results similar for RMFG cluster which overlapped with WMH-RMFG cluster, and for LMFG cluster). In sum, as WMH volume increased, even though there was significantly poorer search task performance, there was both increased middle frontal gyrus activation bilaterally (especially in RMFG) and associated increases in interhemispheric BSCs between homologous frontal regions. Further, we found that WMH burden is associated with WMH-RMFG activation independent of performance (*p* = .00287), supporting the argument that greater WMH volume could be involved in increased but ineffective frontal activation during engagement of attentional control.

## Discussion

In this study we investigated the impact of white matter structural differences as measured by WMH on activation and task-based functional connectivity of a visual attentional control network. We replicated our previous finding that WMH are associated with poorer visual search performance (RT) even in ostensibly “normal” older adults. We hypothesized that OA performing the task would demonstrate attention network node activation, and that increasing volumes of WMH in OA would be inversely associated with the extent and magnitude of activation within these nodes as previously noted [8]. Further, we hypothesized that the extent of WMH would be associated with decreased functional connectivity. While we confirmed the expected reduction in search performance with extent of WMH [9], we unexpectedly found that greater WMH volume was associated with greater fMRI activation during visual search in brain regions important to top-down and bottom-up attentional control. This included a significant cluster in the RMFG (Fig. 3b) where greater WMH volume was associated with increased activation with linear set size increases among the OA group. Even more surprisingly, we found that greater OA WMH volume independently predicted selectively increased frontal interhemispheric functional connectivity. Not only was increased frontal interhemispheric functional connectivity associated with increased middle frontal activation among elders, but the increased functional connectivity also was significantly, inversely related to visual search performance. This, combined with a finding that WMH volume is associated with RMFG activation independent of performance, could suggest that greater WMH volume is involved in increased but ineffective frontal activation during engagement of attentional control. These results suggest that CVD- and inflammation-related processes like WMH might impact cognitive network functional profiles by a specific mechanism distinct from the aging process, and demonstrates that white matter structural alterations, particularly of frontally anchored networks, are associated with dysfunctional patterns of brain activation, functional connectivity and cognitive performance.

It is recognized that older adults exhibit greater bilateral activation of brain networks (i.e., Hemispheric Asymmetry Reduction in OLDer adults or HAROLD model [20]), and a posterior to anterior shift in activation [55]. Therefore, while older adults tend to activate more regions in general, they demonstrate less connectivity between anterior and posterior brain regions. In some cases, the increased activation is associated with improved performance; however, in others (such as ours) it is associated with poorer performance. Until now, there has been little evidence to explain why this may occur. Results from our study using a visual search task confirmed that older adults activate bilateral brain networks, and our results may also suggest that increased WMH burden also leads to increased activation and poorer performance. This might be consistent with models of brain network dedifferentiation and inefficiency [21], as these results indicate that in response to white matter structural differences (WMH), and independent of actual subject chronological age, brain networks activate less selectively. As WMH volume increases, so does activation of frontal cortex and interhemispheric BSC, but critically, task performance declines. Such a finding supports the hypothesis that WMH lead to greater “noise” in the system (i.e., noisier inputs from posterior processing regions into frontal cortex) resulting in greater burden to cortical systems to process noisy information, and consequently less effectual activation of cortical (particularly frontal) systems [56]. WMH have been suggested to be associated with diffuse disconnection of white matter tracts throughout the brain, and that this disconnection process may be a mechanism underlying the disruption of frontal function in control and memory tasks in otherwise cognitively healthy aging [7,8]; the results in the current study extend this idea to include frontal function during attention task performance, although the neurobiological hypothesis of WMH specifically leading to tract disconnection and subsequent functional differences requires further testing.

The brain networks engaged during task performance, particularly those (Fig. 3a, *set level*) networks identified by associations between activation and set size, suggest the coordination of a distributed frontal-parietal attentional control system with visual cortex and subcortical relays. This is indicative of greater engagement of top-down (dorsal) and bottom-up (ventral) attentional control with visual cortex activation, in order to subserve performance with increasing task difficulty in OA subjects.

Our findings, particularly the WMH-related frontal cluster result (Fig. 3b) and functional connectivity results bear importance on cognitive aging theories. Given our results, particularly the associations with performance, it is likely that WMH-related activation increases reflect dedifferentiation or reduced efficiency (i.e., reduced signal-to-noise in neuronal communication) of cortical networks during task performance [21,57] as performance was similarly reduced with greater WMH. WMH-related increased frontal activation and functional connectivity did not appear to serve a successful cognitive purpose, as task performance was inversely associated with frontal interhemispheric BSC magnitude, and as WMH volume predicted activation independent of performance. As such, the current results strongly suggest that the underlying mechanism linking WMH to altered brain function is not compensatory, as network functional activation and connectivity differences are ineffective. The elevated functional activation and connectivity may represent a “toxic consequence” of elevated WMH on network function. While subtle compensatory network reorganization processes might occur in the presence of WMH, they are mechanisms of ineffective, futile over-activation. We propose that WMH exert their effect on cognition through injury and otherwise deleterious change to the structural connections of distributed cognitive networks necessary for specific cognitive functions, such as cognitive control and attentional control, by dedifferentiating and reducing the efficiency of information transfer amongst various parts of these networks, which we observed as futilely increased functional activation and connectivity of frontal network regions while task performance worsened. WMH may lead to greater but less effective activation of cortical systems in attempt to address task demands.

We hypothesize that subjects performing this task send neural inputs from parietal processing regions into frontal control regions, utilizing the available white matter connections between parietal and frontal cortex, and producing a characteristic pattern of functional activation and connectivity. With more WMH, representing more disruption to OA white matter structural connections across the network, there is greater network dedifferentiation and frontal cortex activates less selectively and more bilaterally; as less selective information processing occurs in homologous frontal cortical regions, frontal interhemispheric functional connectivity increases. In this case, increased functional connectivity (measured using correlation coefficients) would signify a decrease in the independence of information processing in homologous frontal regions, as highly correlated signals would be expected if homologous frontal regions were doing nearly identical signal processing, consistent with dedifferentiation theories. Recent research in epilepsy populations, for example, has suggested that increased functional connectivity with greater disease burden could represent functional decoupling between cortical regions, and such functional differences demonstrate reduced network efficiency as they are associated with reduced performance [58].

There are potential limitations to our study. One is that CVD (which is known to be associated with WMH volume) affects neurovascular coupling whether or not it injures neurons, a limitation of all CVD-BOLD studies. However, it is unlikely that WMH-activation associations are primarily driven by co-occurring WMH and neurovascular-coupling differences, as we found WMH-related activation increases in areas engaged during task. Relatively low subject numbers and limited OA age variance may also impair our ability to examine associations between OA chronological age, WMH volume, and function. However, the current experiment was designed not to test effects across a wide age range, but to test functional profiles across a WMH volume range characteristic of cognitively healthy elders.

## Conclusions

Greater WMH volume among older adults is associated with differences in task-based functional activation and connectivity during visual search in regions important to both top-down and bottom-up attentional control. WMH may lead to subtle attention network dedifferentiation and dysfunction independent of chronological age alone, altering frontal lobe functional activation and connectivity during task performance. Our results suggest that there are some components of frontal-parietal activation amongst older individuals, particularly those important to cognitive control, that are likely related to clinically asymptomatic, vascular- and inflammation-related white matter structural connectivity alterations called WMH [59,60], and are not just due to advanced age alone. This might indicate, given the known strong association of WMH with CVD, a prodromal stage of more advanced CVD where treatment of cardiovascular risk factors may be most useful. The specific impacts of WMH on cognition and network functional activation and connectivity in ostensibly healthy aging have received little study, yet are crucial to understanding mechanisms of cognitive decline. Our results confirm a growing literature that suggests that some normal age-related cognitive differences may be due to clinically asymptomatic white matter differences. Early, aggressive treatment of risk factors may deter further brain changes and cognitive decline; therefore, there is great public health benefit to investigating the contribution of CVD and inflammation to cognitive decline in healthy aging.

## Supporting Information

S1 Table OA Activation clusters and peak coordinates for *Set level* contrast. (DOC)

S1 Fig FMRI Activation results by age group and WMH volume using study-specific template. Top panel: Standard MNI and study specific EPI templates. Middle panel: Activation for older adults for *set level* search contrast; clusters labeled as significant if passing a *p* <. 001 uncorrected voxel height threshold with 10 voxel cluster extent. Bottom panel: *Set level* activation significantly associated with WMH volume among OA; clusters labeled as significant if passing a *p* <. 005 uncorrected voxel height threshold with 10 voxel cluster extent.
(TIFF)
