# Supplementary material for: White Matter Hyperintensities among Older Adults Are Associated with Futile Increase in Frontal Activation and Functional Connectivity during Spatial Search
Source: PLoS One. 2015 Mar 20;10(3):e0122445. doi: 10.1371/journal.pone.0122445 (PMC4368687; doi:10.1371/journal.pone.0122445)
Supplement: S1 Table — (DOC) [file pone.0122445.s001.doc]

Table S1

OA Activation clusters and peak coordinates for *Set level* contrast.

Cluster size *T* values *X Y Z* Location

4957 9.94 27 -64 46 R superior parietal lobule (BA 7)

9.82 21 -100 19 R middle occipital gyrus (BA 18)

9.36 -18 -67 40 L precuneus (BA 7)

8.51 9 -97 7 R cuneus (BA 18)

8.32 -27 -73 28 L precuneus (BA 31)

8.30 -24 -70 31 L precuneus (BA 7)

8.24 -6 -97 -5 L lingual gyrus (BA 17)

8.15 36 -79 19 L middle temporal gyrus (BA 19)

8.14 33 -76 22 R superior occipital gyrus (BA 19)

7.98 18 -82 -14 R lingual gyrus (BA 18)

7.93 9 -85 -8 R lingual gyrus (BA 18)

7.91 -21 -85 -17 L fusiform gyrus (BA 19)

7.63 -33 -94 13 L middle occipital gyrus (BA 19)

7.49 -30 -85 16 L middle occipital gyrus (BA 19)

7.34 -18 -103 16 L middle occipital gyrus (BA 18)

7.26 -21 -100 19 L middle occipital gyrus (BA 18)

192 6.82 33 23 -2 R insula (BA 13)

481 6.76 -45 5 31 L inferior frontal gyrus (BA 9)

5.50 -27 -1 52 L middle frontal gyrus (BA 6)

4.73 39 -7 43 R precentral gyrus (BA 6)

3.98 -51 26 28 L middle frontal gyrus (BA 46)

381 6.73 6 -25 -8 R thalamus

6.14 12 -1 -2 R globus pallidus

6.01 -6 -25 -8 L red nucleus (midbrain)

5.55 -12 -4 -5 L lentiform nucleus/globus pallidus

4.99 12 -13 -11 R subthalamic nucleus

3.68 3 -25 -26 R midbrain

489 6.13 42 11 28 R middle frontal gyrus (BA9)

5.10 -39 -7 43 L precentral gyrus (BA 6)

5.08 45 23 28 R middle frontal gyrus (BA 9)

5.08 27 8 49 R middle frontal gyrus (BA 6)

5.04 42 -4 46 R middle frontal gyrus (BA 2)

4.65 54 29 31 R middle frontal gyrus (BA 9)

3.93 45 35 37 R middle frontal gyrus (BA 9)

91 5.28 -30 23 -5 L insula/inferior frontal gyrus (BA 13/47)

3.67 -27 20 -20 L inferior frontal gyrus (BA 47)

36 5.15 -9 14 52 L medial frontal gyrus (BA 8)

39 4.66 9 17 52 R medial frontal gyrus (BA 8)

13 4.15 12 62 28 R superior frontal gyrus (BA 10)

3.70 18 62 25 R superior frontal gyrus (BA 10)
