# Supplementary figures and images for: White Matter Hyperintensities among Older Adults Are Associated with Futile Increase in Frontal Activation and Functional Connectivity during Spatial Search
Source: PLoS One. 2015 Mar 20;10(3):e0122445. doi: 10.1371/journal.pone.0122445 (PMC4368687; doi:10.1371/journal.pone.0122445)

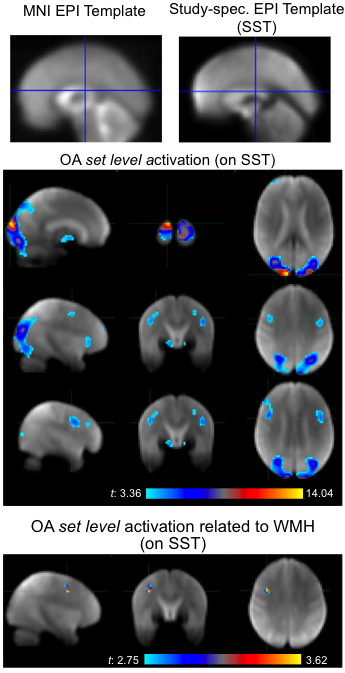

Supplement: S1 Fig — Top panel: Standard MNI and study specific EPI templates. Middle panel: Activation for older adults for set level search contrast; clusters labeled as significant if passing a p <. 001 uncorrected voxel height threshold with 10 voxel cluster extent. Bottom panel: Set level activation significantly associated with WMH volume among OA; clusters labeled as significant if passing a p <. 005 uncorrected voxel height threshold with 10 voxel cluster extent. (TIFF) [file pone.0122445.s002.tiff]
